# The Institutional Worker

**Published:** 1913-04-19

**Authors:** 


THE
### INSTITUTIONAL WORKER
Being a Special Supplement to "The Hospital"

### The Organisation of Workshop Collections.

Success in the periodical collection of funds at workshops and factories rests not so much upon the method of collection adopted as upon the enthusiasm of the workpeople. You should call upon the employers of labour in your district and obtain their permission to speak to their employees upon the subject of your institution. If you are successful in arousing interest and in securing the services of a responsible person amongst each body of workpeople to look after the weekly collections, you should have no further difficulty.—"Pence."

### Visitors to Patients.

The responsibility of admitting visitors to patients in the wards of a hospital rests with the honorary medical staff or their representative; and although an arrangement may be made between the staff and yourself in this matter, for your mutual convenience, it is important in the interests of your hospital that there should be no interference with this responsibility.—M.

### Where to get C.M.B. Training.

A coupon should be enclosed with each inquiry; will you please send it on? To save time we give you the information you ask for. There are many excellent maternity schools in London and the large towns throughout the country. Though a London training certainly does carry weight in some quarters, more especially in general nursing, this question is not of paramount importance. If it would be as convenient for you to train in London as elsewhere, apply to one of the lying-in charities, such as Queen Charlotte's Lying-in Hospital, Marylebone Road, London, N.W. If you desire to get an appointment in the Colonies you should apply to the Colonial Nursing Association, Imperial Institute, London, S.W.—N. R.

### How to Become a Health Visitor.

There is no reason at all why you should not enter for the Health Visitors' Examination of the Royal Sanitary Institute; the only restriction is that candidates must be over twenty-one years of age. It is, of course, competent upon the various local authorities engaging health visitors, school nurses, etc., to fix an age limit, but this is by no means an invariable rule, and your age should not interfere with your success in obtaining a post. Write to the Secretary of the Institute, Buckingham Palace Road, S.W. — "Anxious."

### Starting a Special Home.

The information you give is somewhat too scanty for us to advise you properly. The terms you should fix must necessarily depend upon local conditions, upon the number of patients you are prepared to accommodate, and as to whether nursing attendance is to be included. In some of the good provincial towns from £5 5s. to £10 10s. a week is charged for cases such as you propose to take, varying in accordance with the nature of each case and the accommodation afforded. We keep a Register of Approved Homes, and if when you have made your arrangements you would care to comply with our conditions we should be pleased to help you through this department.—G. E.

### Country Holidays for Poor Children.

The Children's Country Holidays Fund allows the weekly sum of 5s. to cover the cost of board, lodging, and washing of each child. If, as you say, you are fond of children, and are desirous of assisting each year a few of the ailing children in the poor quarters of the Metropolis, you should communicate with the Secretary of this Fund at 18 Buckingham Street, Strand, W.C., who will arrange for the Country Correspondent of your district to call upon you. The greatest care is taken by the Society's voluntary workers that the children are sent away clean and free from infectious disease. The holiday of each child is limited to a fortnight, except in special circumstances, as convalescence from illness. The aim of the Society is to give the children a really good holiday, such as will not only improve their physical condition, but will also, by arousing the interest of the child mind in the beauties of the countryside, widen their view of life, and give them the knowledge and hope of something better. The Countryside Committee attached to the Society is doing much toward furthering this aim, but the co-operation of "cottage hostesses" is indispensable, so that you would have ample opportunities of fulfilling your wishes in this direction.—"Parkstone."

### OUR BUREAU OF INFORMATION.

### Rules for Correspondents.

1. Every letter, on whatever subject, must be accompanied by the coupon to be cut from the back cover (inside page) of The Hospital, current issue, and must contain the name and address of the correspondent with pseudonym for publication if desired.
2. Replies by post cannot be given, save under exceptional circumstances at the Editor's discretion.
3. Letters from Approved Homes in reply to special needs published in the Bureau should state terms and full particulars, and be sent prepaid under cover to the Editor of the Bureau with name written across coupon for identification.
4. Proprietors of Homes which have not yet been entered on the List of Approved Homes, but have spare accommodation likely to suit special needs, are invited to write for an application form for registration. The fee for registration, which includes two announcements of the Home in the Bureau and other privileges, is 10s.
5. All communications to be addressed to the Editor of The Hospital, 28 Southampton Street, Strand, London, W.C., and marked "Bureau of Information."

### Invitation to Readers.

**The Editor is prepared to make known without charge the needs of any who may be sick or in difficulties, and to guide them in making choice of Homes for treatment or convalescence. Reduced terms can often be arranged with Homes on the Approved List for those unable to pay full fees. Queries are specially invited from those who are engaged in any kind of philanthropical work. A new edition of the leaflet describing the purposes of the Bureau of Information is now ready and will be sent free of charge on application.**

### An Interesting Point for Maternity Nurses.

An obstetrical nurse agreed to attend a case at a reduced fee of £8 8s. for the month and her usual fee of £2 2s. a week for extra time. The nurse was accordingly engaged, and took up duty on the appointed date. She awaited the confinement for a fortnight, and, as it then appeared that there might be further delay, she, at the request of her employer, agreed to return and to wait at her own home. This she did, leaving behind, at her employer's suggestion, the bulk of her luggage and her nursing requisites. She verbally agreed to charge a waiting fee only if she were compelled to refuse other work, and her employer arranged to notify her as soon as her services were required. A month later she was sent for, and, responding to the call, she fulfilled her engagement, which, in accordance with the arrangement made at her employer's request, rendered it necessary for her to sacrifice one week of her engagement in connection with her next booking. The nurse was also constrained to refuse night work during the period of waiting at her own home.

### The Legal Position.

The employer declines to admit that any waiting fee is due, and disbelieves the nurse's statement respecting her refusal of other work. After paying the fee of £8 8s. he offers £2 2s. in settlement of all other obligations, which the nurse refuses. The facts in the above case are important, and it is unfortunate that they are

### Pensions for Domestic Servants.

The Domestic Servants' Benevolent Institution provides pensions for domestic servants who, being incapable of active duty, are members of the Institution by virtue of a small annual subscription or an equivalent life subscription. The Institution also grants relief in urgent cases of temporary distress. Full particulars may be obtained of the Secretary, 199 Piccadilly, London, W.—"Mistress."

### Help for Discharged Prisoners.

The Holloway Discharged Prisoners' Aid Society will help you. Write to the Secretary, 117 Victoria Street, London, S.W., who will put you into communication with the Society's agent located in your neighbourhood. The Society befriends and assists women upon their discharge from Holloway Prison, and prisoners are visited in the prison in order that the agents may know how best to assist them upon their discharge. A few of the friendless and destitute are temporarily accommodated in the "House of Help," which is supported by the Society, until suitable situations can be found for them.—"Troubled."

### Antiseptic Cleaning Material.

The name of the white antiseptic powder you refer to is probably "Gospo"; it may be obtained at most general stores in tins at 4½d. each, but you could obtain prices for larger quantities from the manufacturers, Gospo, Ltd., 33 Waterloo Road, London, S.E.—L. W.

### Scarcity of Private Nurses.

You are by no means singular in the difficulty you experience, which is a growing one. It is possible that your advertisement does not sufficiently justify the character of the posts you have to offer. A well drafted advertisement in *The Nursing Mirror* ought to help you. Have you applied to the Poor-Law infirmaries? You can obtain excellent medical and maternity nurses from these institutions. The Matron of the National Hospital, Queen Square, Bloomsbury, London, might be able to help you to obtain the services of trained nurses also qualified as masseuses.—E. A. S.

### Laymen as Radiographers.

It is by no means an invariable rule for appointments as radiographers to be filled by medical practitioners, although when a layman is employed in this capacity at a hospital he acts under the direction of the medical staff, who are primarily responsible for the work he does. In the large hospitals laymen are employed as assistants, and the pay varies from 30s. to £2 a week. If you desire to obtain such an appointment you should advertise your want, and reply to the advertisements which occasionally appear in the medical journals. If you are in touch with medical men prepared to support you, and have sufficient capital, you could do better by starting in rooms on your own account, in which case you should deal only with patients referred to you by the medical practitioners supporting you.—W. H. McC.

not recorded in writing. The material points to be borne in mind in considering the question submitted are whether at the time of the engagement the exact terms of the nurse's remuneration were clearly conveyed to the mind of the employer, and, further, when the nurse was requested to wait at home, whether it was clearly understood by the employer that she would charge for her time while waiting if she had to refuse any cases. The nurse would have to prove that she did refuse cases, and she can only claim for the time which would have been occupied by such cases had she not refused them. The fact that she was requested to leave her uniform, etc., with her employer is strong evidence of the fact that the employer considered her still engaged to her while waiting at home. It is not stated whether the remuneration enumerated included board and lodging; possibly a claim for this might be made in respect of the period while waiting for the case at home.—"E. B."

### A Hostel for Working Lads.

Apply to the Superintendent, "The Grotto Home," 55 Paddington Street, W. There are always a certain number awaiting admission, and it is improbable that the lad, if approved, could be admitted at once, but it is desirable that you should have his name placed on the waiting list as soon as possible. The Home was established to provide board and lodging, with helpful Christian influences, for poor lads between 14 and 17 years of age, just starting out in life, and having or desiring suitable employment. Board includes three good meals a day, and the boys are expected to contribute toward the expense of their maintenance.—"District Visitor."

### Defective Cathodal Tubes.

The cause of the trouble is due to a perforation of your tube, and the only remedy is re-exhaustion. Any reputable agent dealing in *x*-ray apparatus will get this done for you. Ask your agent to put you in communication with the makers; you could then send on your defective tubes and get a report from them direct. See that the leads from the coil do not cross the tube; neglect of this precaution is frequently the cause of damage. The proper use and care of cathodal tubes is too wide a subject to deal with here, but if you will write to The Scientific Press, Ltd., they will forward you a list of useful books upon this subject.—X. Y. Z.

### Institution Facts and Figures.

### April Question.

Stating the number of beds in your institution and the class of patients received, give the following particulars:—

The number of garments washed during any four weeks in the winter months, with the price per unit, and the manner in which the cost is arrived at. In addition to wages, fuel, light and power, interest on capital should be taken into account. State the quantity, description, and cost of soap used in laundry for the same month. If curd and oil soap be used, the amount of alkali should also be stated. State also the quantity of starch and blue.

The best answers will be published, and for each contribution considered by the Editor to be of sufficient interest for publication payment of Five Shillings will be made.

All letters to be addressed, with coupon enclosed, to The Editor, The Bureau of Information, 28-29 Southampton Street, Strand, London, W.C., and to be received by the last day of the current month.

### Hospital Report.

You can obtain the report you refer to by applying to the Secretary of the Royal Infirmary, Leicester. — "Boston, L. H. B."

### The Ethics of Courtship and Marriage.

An enormous amount is published every year on this subject, and without referring you to the innumerable American publications, often of doubtful merit, which can be obtained everywhere, perhaps the most scholarly study of recent years is contained in the sixth volume of Havelock Ellis' "Studies in the Psychology of Sex," Lippincott and Co., which, amongst others, may be obtained through The Scientific Press, Ltd., 28-29 Southampton Street, Strand, London, W.C.—Elum.

### The Editor's Letter-Box.

### Communications have been received and attended to from:—

M. Aldridge, M. E. A. Martin, Nurse Green, Mrs. Grunnewood, B. Arnold, Edith M. Wheeler, Mrs. Marden, and Sister A. C. Jennings Goodley.

L. Starey.—We have forwarded your letter to our correspondent.

F. R. Ward.—We only forward letters from Approved Homes. The information you give is useful, and the Homes are likely to meet the requirements of our readers. We should be glad to place you on our Register of Approved Homes, and we are forwarding the conditions of registration.

### Editor's Notices.

**Contributions:**

Contributions should be written, or preferably typed, on one side of the paper only, and all articles sent in are accepted upon the distinct understanding that they are forwarded to The Hospital only.

The Editor cannot undertake to return MSS. not used, but when a stamped directed envelope is enclosed the MSS. may be returned if a special request is made.

Accepted articles and paragraphs of news will be paid for after publication at the scale rate.

**Address:**

To prevent delay all contributions and letters on editorial business must be addressed exclusively to the Editor, "The Hospital," 29 Southampton Street, Strand, London, W.C. It is important that this regulation shall be strictly observed.

**Correspondence:**

Correspondence on all subjects is invited. The name and address of correspondents must be given as a guarantee of good faith, but not necessarily for publication.

**Special Articles:**

Special articles are invited, and questions, inquiries, and paragraphs upon all matters relating to the work, administration, and management of general, special, mental, fever, cottage and convalescent hospitals, sanatoria, homes, institutions, societies and organisations for the treatment or care of the sick, injured and dependants of all classes. Every matter affecting the interests and welfare of the staff of all grades working in these institutions will receive special consideration.

**Administrative Medicine, Research and Items of News:**

Special terms will be paid for approved articles on subjects in this wide field by experts who have made some section of it their special study and interest. We wish to give prominence to every movement tending to advance accuracy of diagnosis, efficiency in treatment, and the development of modern methods to eradicate disease and promote the welfare of the suffering.

**A Bureau of Information:**

We invite inquiries and applications for information and help in providing for that numerous class of sufferers who require special care or house-room which they cannot obtain in their own homes or secure for themselves. We cannot, however, prescribe, or recommend practitioners

**Books for Review:**

Publishers are particularly requested to send advance proofs of any new books of importance whenever possible, as the Editor has made arrangements to publish immediate reviews on a new plan.

**Photographs, Plans, Blocks, and Illustrations:**

It is requested that wherever possible MSS. may be accompanied by illustrations in any of the above forms. The name of the sender and of the article to which it belongs should be written on the back of each photograph, drawing, or block for purposes of identification.

**Local Papers:**

Newspapers containing reports or news paragraphs should be marked and addressed to the Sub-Editor.

**The Coupon System:**

A coupon will be found at the bottom of the third inside page of the cover each week. This coupon must be attached to every question or inquiry to which an answer is desired in The Hospital.

### A Help to the Worker.

As a means of announcing vacancies in every department of Institutional work, the success of *The Institutional Worker* is evidenced by the ever-increasing number of advertisements that appear in this section of The Hospital. No more adequate proof of the value of *The Institutional Worker* can be desired than the fact that Authorities of the more important Institutions throughout the United Kingdom employ its columns again and again whenever they have a vacancy to fill upon their staffs. Moreover, it provides exceptional facilities for every class of announcement affecting Institutional Life and Work, and as a medium for securing appointments in any section of the Institutional, Health, and Sanitary Services it is a distinct help to the worker, since no other journal possesses such a comprehensive circulation in these directions as The Hospital. If you are seeking a post an announcement of twenty-one words, costing the small sum of 1s., will save you many hours of anxious thought, and certainly bring your requirements immediately to the notice of those most likely to need your services.

The form below may be used for Appointments Wanted advertisements, and when filled up should be posted to the

*Manager*, The Hospital,
28 and 29 Southampton Street,
Strand, W.C.

Appointments Wanted, 21 words ... 1s. 0d.

| | | |
|---|---|---|
| | | |
| | | |
| | | |
| | | |
| | | |
| | | |
| | | |

### Manager's Notices.

**Letters relating to the publication, sale, and advertising departments of "The Hospital" must be addressed to The Manager, "The Hospital," The Hospital Building, 28 and 29 Southampton Street, London, W.C.**

**Advertisements:**

To ensure insertion, all advertisements for The Hospital must reach the Manager not later than Wednesday morning in each week. For scale of charges see pages iii and 4.

**Subscriptions** may begin at any time, and are payable in advance. Cheques and Post-Office Orders should be crossed London County and Westminster Bank, Covent Garden Branch, and made payable to the Manager of The Hospital.

Rates of Subscription (including Postage).

| *United Kingdom.* | | *Foreign and Colonial.* | |
|---|---|---|---|
| Three Months ... ... | 2s. 0d. | Three Months ... ... | 2s. 6d. |
| Six Months ... ... | 4s. 0d. | Six Months ... ... | 5s. 0d. |
| Twelve Months ... ... | 6s. 6d | Twelve Months ... ... | 8s. 8d. |

**Remittances:**

**It is especially requested that remittances be made by Cheque or Postal Order, and in halfpenny stamps only when the amount is under sixpence.**

